# Supplementary material for: Development and validation of scales for speaking self-efficacy: Constructs, sources, and relations
Source: PLoS One. 2024 Jan 29;19(1):e0297517. doi: 10.1371/journal.pone.0297517 (PMC10824441; doi:10.1371/journal.pone.0297517)
Supplement: S1 Appendix — (DOCX) [file pone.0297517.s001.docx]

**S1 Appendix. Interview procedure**

During the item generation phase, semi-structured interview was administered to a total of 10 students. participants were asked to respond in their first language (Chinese). To analyze the transcribed interviews, the data were analyzed by the first author by omitting data which were not relevant to speaking self-efficacy. They then compared their lists of items and together created a 30-item questionnaire.

**Interview protocol sample**

1. Could you please rate your English-speaking proficiency?

2. What do you focus on when speaking English in the classroom?

3. Do you pay attention to the fluency, accuracy, and complexity of your expressions when speaking English in the classroom?

4. Do you think ahead when speaking English in the classroom?

5. Do you interact with the audience when speaking English in the classroom?

6. How do you feel when speaking English in the classroom?

7. Do you practice English speaking outside of the classroom?

8. Would you kindly assess your own English-speaking performance in class?

**Part of the interview transcriptions**

**Participant A:** I find my spoken English to be quite mediocre. I greatly admire those around me who have excellent oral skills. We often have opportunities to practice speaking in class, such as presentations and speeches. To improve my speaking ability, I actively participate during class and practice with friends after class. Initially, I would feel nervous, but with more practice, the nervousness reduces. Before speaking, I mentally draft my thoughts in English, without considering Chinese. However, I struggle to pay attention to the audience and mostly focus on expressing myself. I try to perform well in various aspects of my spoken English. I really value grammar and accuracy of sentences that I spoke. If I notice errors I make, I correct them immediately.

**Participant B:** I perceive my oral proficiency to be quite poor. I lack confidence in my speaking abilities. I am unwilling to speak up in class. However, to prepare for the TOEFL, I still practice my oral English with friends after class. Being called upon in class happens infrequently, but when it does, I first think of a sentence in Chinese and then translate it into English. Speaking in English makes me feel extremely nervous. While speaking, I pay attention to the audience and if I see any confused expressions, I repeat myself... I constantly fear making grammar and tense errors, causing my spoken words to lag behind my thoughts.
