# Supplementary material for: Development and validation of scales for speaking self-efficacy: Constructs, sources, and relations
Source: PLoS One. 2024 Jan 29;19(1):e0297517. doi: 10.1371/journal.pone.0297517 (PMC10824441; doi:10.1371/journal.pone.0297517)
Supplement: S2 Appendix — (DOCX) [file pone.0297517.s002.docx]

**S2** **Appendix.** **EFL speaking self-efficacy scale (EFL-SSES)**

| Strongly Disagree | Disagree | Slightly Disagree | Uncerntain | Slightly Agree | Agree | Strongly Agree |
| --- | --- | --- | --- | --- | --- | --- |
| 非常不同意 | 不同意 | 有点不同意 | 不确定 | 有点同意 | 同意 | 非常同意 |
| 1 | 2 | 3 | 4 | 5 | 6 | 7 |

*Linguistic Self-Efficacy (LSE)*

1. When speaking English in the classroom, I can speak fluently. 在课堂上用英语发言时，我可以流利地表达自己的想法。

2. When speaking English in the classroom, I can logically organize my words. 在课堂上用英语发言时，我可以有逻辑地组织自己的想法。

3. When speaking English in the classroom, I can speak with few pause or filler (i.e., “Um,” “Ah,” or “You Know”). 在课堂上用英语发言时，我很少有停顿或磕绊（如：“嗯”、“啊”）。

4. When speaking English in the classroom, I can speak with grammatical accuracy. 在课堂上用英语发言时，我的语法都是正确的。

5. When speaking English in the classroom, I can speak with correct pronunciation, intonation, and liaison. 在课堂上用英语发言时，我的发音、语调和连读都是正确的。

*Self-Regulatory Efficacy (SRE)*

6. I actively participate in my speaking course to improve my speaking. 在课堂上，我会积极把握用英语发言的机会。

7. When speaking English in the classroom, I can think of my goals before speaking. 在课堂上用英语发言前，我已经构思好自己的内容。

8. When speaking English in the classroom, I can evaluate whether I achieve my goal in speaking. 在课堂上用英语发言时，我可以评估是否向听众传递了自己的想法。

*Delivery Self-efficacy (DSE)*

9. When speaking English in the classroom, I can speak with confidence. 在课堂上用英语发言时，我非常自信。

10. I am not stressed out when speaking English in the classroom. 在课堂上用英语发言时，我没有压力。

11. I enjoy speaking English outside the classroom. 在课堂外，我乐意和他人用英语交流。

*Performance Self-Efficacy (PSE)*

12. I can understand the most difficult material presented in speaking course. 我可以理解并掌握口语教学材料。

13. I can do an excellent job on the assignments and tests in the speaking course. 我能够出色地完成口语作业和测试。

14. Considering the difficulty of the speaking course, the teacher, and my skill, I think I can do well in this class. 考虑到口语学习的难度、老师的教学方法和我的能力，我认为我可以在这门课上做得很好。

15. I can receive an excellent grade in speaking course. 我能够在口语考试中获得优秀的成绩。
