# Supplementary material for: Development and validation of scales for speaking self-efficacy: Constructs, sources, and relations
Source: PLoS One. 2024 Jan 29;19(1):e0297517. doi: 10.1371/journal.pone.0297517 (PMC10824441; doi:10.1371/journal.pone.0297517)
Supplement: S3 Appendix — (DOCX) [file pone.0297517.s003.docx]

**S3 Appendix. EFL sources of speaking self-efficacy scale (EFL-SSSES)**

| Strongly Disagree | Disagree | Slightly Disagree | Uncerntain | Slightly Agree | Agree | Strongly Agree |
| --- | --- | --- | --- | --- | --- | --- |
| 非常不同意 | 不同意 | 有点不同意 | 不确定 | 有点同意 | 同意 | 非常同意 |
| 1 | 2 | 3 | 4 | 5 | 6 | 7 |

*Mastery Experience (ME)*

1. In the past, when speaking English in the classroom, I expressed my ideas fluently. 我曾经在课堂上流利地用英语表达我的想法。

2. In the past, when speaking English in the classroom, I spoke all words with correct pronunciation, intonation, and liaison. 我曾经在课堂上用正确的发音、语调和连读讲英语。

3. In the past, I did well on Spoken English assignments. 在以往的口语作业中，我有着良好的表现。

4. In the past, I got excellent grades on Spoken English tests. 在以往的口语考试中，我有着很高的成绩。

*Vicarious Experience (VE)*

5. When I see how my English teacher uses complex sentences, I can picture myself using complex sentences in the same way. 当我看到英语老师能够准确地使用复杂的句式时，我可以想象自己用同样的方法表达自己的想法。

6. When I see how another student logically expresses their ideas, I can see myself logically expressing my ideas in the same way. 当我看到其他同学能够有逻辑地用英语表达自己的想法，我可以想象自己用同样的方法表达自己的想法。

7. When I see how my peers speak with perfect pronunciation and intonation, I can see myself speaking with perfect pronunciation and intonation in the same way. 当我看到我的朋友在英语课上表现出较好的语音语调，我很佩服他/她。

*Social Persuasion (SP)*

8. My teachers have told me that I have a talent for speaking English. 我的老师告诉我，我在英语口语方面很有天赋。

9. My parents have told me that I am doing well in speaking English. 我的父母告诉我，我在英语口语方面做得很好。

10. My classmates have told me that I am good at speaking English. 我的同学告诉我，我很擅长英语口语。

*Physiological and Emotional States (PES)*

11. When speaking English in the classroom, I felt nervous. 当在课堂上用英语发言的时候，我感到紧张。

12. When speaking English in the classroom, I got stressed. 当在课堂上用英语发言的时候，我感到压力。

13. When speaking English in the classroom, I got anxious. 当在课堂上用英语发言的时候，我感到焦虑。
