# Supplementary material for: Development and validation of scales for speaking self-efficacy: Constructs, sources, and relations
Source: PLoS One. 2024 Jan 29;19(1):e0297517. doi: 10.1371/journal.pone.0297517 (PMC10824441; doi:10.1371/journal.pone.0297517)
Supplement: Attachment — Submitted filename: Response letter.docx [file pone.0297517.s005.docx]

**Comments**

We recognize that you obtained ethical approval from your local ethics committee after you began the study: your ethics approval document is dated 08.12.2022, while participants were recruited beginning on 1st December 2022. For human subjects research, *PLOS ONE* requires that authors obtain ethics committee approval before beginning the study, and we do not accept retrospective ethical approval.

Jayati Bhattacharyya, PhD

Editorial Office

**Response**

Thank you for pointing out the typo in our manuscript. The actual data collection date falls after our ethics approval. That is, **11 December 2022**. We have corrected the typo in our revised submission. We fully understand the purpose of obtaining ethics approval which is to protect participants’ rights, well-being, and privacy. That is why we sought ethics approval before initiating our research. We are sorry for the inconvenience caused during the process of our submission.

Authors
