# Supplementary material for: Development and validation of scales for speaking self-efficacy: Constructs, sources, and relations
Source: PLoS One. 2024 Jan 29;19(1):e0297517. doi: 10.1371/journal.pone.0297517 (PMC10824441; doi:10.1371/journal.pone.0297517)
Supplement: Attachment — Submitted filename: Reviewer Message.docx [file pone.0297517.s007.docx]

Dear Authors,

I extend my gratitude for crafting a manuscript that introduces two valuable new instruments. Your efforts are commendable. I really enjoyed reading your work. It's evident that your statistical methods are robust, and the discussion is apt. However, prior to advancing towards publication, a few revisions are in order. These suggestions predominantly pertain to the literature review and methodology sections. I encourage you to refer to the detailed feedback I've shared in the attached manuscript file.

I eagerly await the opportunity to engage with your revised manuscript.

With best regards,
