# Supplementary material for: Development and validation of scales for speaking self-efficacy: Constructs, sources, and relations
Source: PLoS One. 2024 Jan 29;19(1):e0297517. doi: 10.1371/journal.pone.0297517 (PMC10824441; doi:10.1371/journal.pone.0297517)
Supplement: Attachment — Submitted filename: 2.0 Response to comments.docx [file pone.0297517.s009.docx]

29 December, 2023

***PLOS ONE***

Dear Editor,

Thank you very much for giving us the opportunity to refine our paper. We would also like to express our gratitude to the reviewers for their constructive feedback, which has greatly contributed to improving the quality of our paper. We have carefully addressed the reviewers’ comments and suggestions, and made the necessary revisions accordingly. Please find below our responses to the reviewers’ comments.

Best regards,

Authors

**Reviewer #3:**

Thank you for the revised file. I am happy with the revised file. The authors can proofread their paper.

**Our response:** Thank you very much for taking time to review our paper and your previous constructive feedback.

**Reviewer #4:** Dear Authors,

I have thoroughly reviewed your manuscript titled "Development and validation of scales for EFL speaking self-efficacy: Constructs sources and relations." Your study offers valuable insights into the area of EFL speaking self-efficacy and contributes significantly to the existing body of knowledge. However, there are several areas where the manuscript could be strengthened.

**Our response:** Thank you for the recognition of our paper.

1. Consistency in Terminology: Maintain consistent use of key terms throughout the paper. For example, terms like "self-efficacy," "speaking self-efficacy," and "EFL speaking self-efficacy" should be used consistently to avoid confusion. It is recommended that you refer to this article when it comes to the concept of self-efficacy.

Han, Y., & Wang, Y. (2021). Investigating the correlation among Chinese EFL teachers’ self-efficacy, reflection, and work engagement. Frontiers in Psychology 12. 763234. https://doi.org/10.3389/fpsyg.2021.763234.

**Our response:** Thank you for this valuable comment. We have changed the term “EFL speaking self-efficacy” to “speaking self-efficacy” to avoid confusion. We have added the recommended study in the literature review.

2. Comprehensiveness: Ensure that the literature review covers all relevant studies, especially recent research, to demonstrate the current state of knowledge in the field. Instead of only summarizing previous studies, critically evaluate them to highlight their contributions and limitations in relation to your research.

**Our response:** Thank you this valuable comment. We have added more recent research to update the literature review. Additionally, we re-organized and re-wrote the literature review concerning previous studies with critiques to rationalize our current study. Please find the highlighted Section 2.2 and Section 2.3 for our revision.

3. Methodology Section: Provide more detail on the sampling strategy to clarify how representativeness was ensured or the limitations. In the item generation and validation process, consider elaborating on how you ensured the cultural relevance of the items, especially since the study focuses on Chinese EFL learners.

**Our response:** Thank you for the feedback. We have added details on the sampling strategy in the Participants parts of Section 3.1.2, Section 3.1.3, Section 3.2.2, and Section 3.2.3 for clarification. Please find the highlighted in Section 3.1.2, Section 3.1.3, Section 3.2.2, and Section 3.2.3 for details.

We thank the reviewer for this valuable comment. In order to ensure the cultural relevance of items, we have elaborated on the translation and pilot tests during item generation process. Please find the highlighted in Section 3.1.1 and Section 3.2.1 for details.

4. Limitations section: Acknowledge the limitations of your study more explicitly, such as the reliance on self-report measures, which might introduce response biases. This study focuses on a specific population (Chinese EFL learners) and context, which may limit the generalizability of the findings to other cultural or linguistic backgrounds. You can supplement it in Limitations. It is recommended that you could refer to this article

Derakhshan, A., Wang, Y. L., Wang, Y. X., & Ortega-Martín, J. L. (2023). Towards Innovative Research Approaches to Investigating the Role of Emotional Variables in Promoting Language Teachers’ and Learners’ Mental Health. International Journal of Mental Health Promotion, 25 (7): 823-832. doi:10.32604/ijmhp.2023.029877.

**Our response:** We thank the reviewer for this comment. We have modified the limitations section according to your suggestion. Please find the highlighted in Section 5 (p. 33) for details. We have added this relevant research in the limitation part.

Language and Style: Perform a thorough proofreading to catch any minor grammatical or syntactical errors. Ensure consistency in terminology and style throughout the paper.

**Our response:** We thank the reviewer for the feedback. We have corrected the errors and thoroughly proofread and refined our manuscript.

Overall, your research is promising and with these suggested improvements, it has the potential to make a more impactful contribution to the field of EFL education.
