# Supplementary material for: Development and validation of scales for speaking self-efficacy: Constructs, sources, and relations
Source: PLoS One. 2024 Jan 29;19(1):e0297517. doi: 10.1371/journal.pone.0297517 (PMC10824441; doi:10.1371/journal.pone.0297517)
Supplement: Attachment — Submitted filename: PONE-D-23-22474_with reviewer comments.pdf [file pone.0297517.s006.pdf]

PLOS ONE

Development and validation of scales for EFL speaking self-efficacy: Construct, sources, and relations

--Manuscript Draft--

**Manuscript Number:**	PONE-D-23-22474
**Article Type:**	Research Article
**Full Title:**	Development and validation of scales for EFL speaking self-efficacy: Construct, sources, and relations
**Short Title:**	EFL speaking self-efficacy scales
**Corresponding Author:**	Peijian Sun<br>Zhejiang University<br>Hangzhou, Zhejiang CHINA
**Keywords:**	speaking self-efficacy; factor analysis; scale development; scale validation
**Abstract:**	Speaking is not a compulsory language skill assessed in the English subject of the National College Entrance Examination in China. This explains why, in elementary and secondary schools, less focus has been placed on the development of English-speaking abilities among Chinese students, resulting in their unbalanced mastery of language skills. Although self-efficacy is a crucial factor influencing students' language performance, our understanding of speaking self-efficacy is insufficient in terms of its construct, its sources, and the relationships between the two. We, therefore, constructed psychometrically sound instruments to measure EFL speaking self-efficacy, including the EFL Speaking Self-Efficacy Scale (EFL-SSES) and the EFL Sources of Speaking Self-Efficacy Scale (EFL-SSSES), in accordance with Bandura's self-efficacy theory. Additionally, we performed path analysis to understand the relationship between the construct and the sources of EFL speaking self-efficacy. The results revealed the key role of physiological and emotional states and marginal importance of vicarious experience for speaking self-efficacy, advancing our grasp of self-efficacy theory in the speaking domain. Our research sheds light on how to assist researchers and educators in identifying and enhancing students' speaking self-efficacy via a variety of sources.
**Order of Authors:**	Peijian Sun
	Yawen Wang
**Additional Information:**	
**Question**	**Response**
**Financial Disclosure**<br><br>Enter a financial disclosure statement that describes the sources of funding for the work included in this submission. Review the submission guidelines for detailed requirements. View published research articles from *PLOS ONE* for specific examples.<br><br>This statement is required for submission and **will appear in the published article** if the submission is accepted. Please make sure it is accurate.	The work was supported by the National Social Science Foundation of China awarded to Dr. Peijian Sun (Grant no. 19CYY008, website http://www.nopss.gov.cn/).

Powered by Editorial Manager® and ProduXion Manager® from Aries Systems Corporation

**Unfunded studies**<br>Enter: *The author(s) received no specific funding for this work.*<br><br>**Funded studies**<br>Enter a statement with the following details:<br>• Initials of the authors who received each award<br>• Grant numbers awarded to each author<br>• The full name of each funder<br>• URL of each funder website<br>• Did the sponsors or funders play any role in the study design, data collection and analysis, decision to publish, or preparation of the manuscript?<br>• **NO** - Include this sentence at the end of your statement: *The funders had no role in study design, data collection and analysis, decision to publish, or preparation of the manuscript.*<br>• **YES** - Specify the role(s) played.<br><br>* typeset	
**Competing Interests**<br><br>Use the instructions below to enter a competing interest statement for this submission. On behalf of all authors, disclose any competing interests that could be perceived to bias this work—acknowledging all financial support and any other relevant financial or non-financial competing interests.<br><br>This statement is required for submission and **will appear in the published article** if the submission is accepted. Please make sure it is accurate and that any funding sources listed in your Funding Information later in the submission form are also declared in your Financial Disclosure statement.<br><br>View published research articles from *PLOS ONE* for specific examples.	I have read the journal's policy and the authors of this manuscript haveno competing interests.

**NO authors have competing interests**<br><br>Enter: *The authors have declared that no competing interests exist.*<br><br>**Authors with competing interests**<br><br>Enter competing interest details beginning with this statement:<br><br>*I have read the journal's policy and the authors of this manuscript have the following competing interests: [insert competing interests here]*<br><br>* typeset	
**Ethics Statement**<br><br>Enter an ethics statement for this submission. This statement is required if the study involved:<br><br>• Human participants<br>• Human specimens or tissue<br>• Vertebrate animals or cephalopods<br>• Vertebrate embryos or tissues<br>• Field research<br><br>Write "N/A" if the submission does not require an ethics statement.<br><br>General guidance is provided below. Consult the submission guidelines for detailed instructions. **Make sure that all information entered here is included in the Methods section of the manuscript.**	The study was reviewed and approved by the Human Participants Ethics Committee of School of International Studies at Zhejiang University (Approval No. SIS2022-01). Informed consent was obtained from all the participants involved in the study including their consent for paper publication based on the collected data.

**Format for specific study types**<br><br>**Human Subject Research (involving human participants and/or tissue)**<br>• Give the name of the institutional review board or ethics committee that approved the study<br>• Include the approval number and/or a statement indicating approval of this research<br>• Indicate the form of consent obtained (written/oral) or the reason that consent was not obtained (e.g. the data were analyzed anonymously)<br><br>**Animal Research (involving vertebrate animals, embryos or tissues)**<br>• Provide the name of the Institutional Animal Care and Use Committee (IACUC) or other relevant ethics board that reviewed the study protocol, and indicate whether they approved this research or granted a formal waiver of ethical approval<br>• Include an approval number if one was obtained<br>• If the study involved *non-human primates*, add *additional details* about animal welfare and steps taken to ameliorate suffering<br>• If anesthesia, euthanasia, or any kind of animal sacrifice is part of the study, include briefly which substances and/or methods were applied<br><br>**Field Research**<br><br>Include the following details if this study involves the collection of plant, animal, or other materials from a natural setting:<br>• Field permit number<br>• Name of the institution or relevant body that granted permission	
**Data Availability**<br><br>Authors are required to make all data underlying the findings described fully available, without restriction, and from the time of publication. PLOS allows rare exceptions to address legal and ethical concerns. See the PLOS Data Policy and FAQ for detailed information.	No - some restrictions will apply

A Data Availability Statement describing where the data can be found is required at submission. Your answers to this question constitute the Data Availability Statement and **will be published in the article**, if accepted.<br><br>**Important:** Stating 'data available on request from the author' is not sufficient. If your data are only available upon request, select 'No' for the first question and explain your exceptional situation in the text box.<br><br>Do the authors confirm that all data underlying the findings described in their manuscript are fully available without restriction?	
**Describe where the data may be found in full sentences. If you are copying our sample text, replace any instances of XXX with the appropriate details.**<br><br>• If the data are **held or will be held in a public repository**, include URLs, accession numbers or DOIs. If this information will only be available after acceptance, indicate this by ticking the box below. For example: *All XXX files are available from the XXX database (accession number(s) XXX, XXX.*).<br>• If the data are all contained **within the manuscript and/or Supporting Information files**, enter the following: *All relevant data are within the manuscript and its Supporting Information files.*<br>• If neither of these applies but you are able to provide **details of access elsewhere**, with or without limitations, please do so. For example:<br><br>*Data cannot be shared publicly because of [XXX]. Data are available from the XXX Institutional Data Access / Ethics Committee (contact via XXX) for researchers who meet the criteria for access to confidential data.*<br><br>*The data underlying the results presented in the study are available from (include the name of the third party*	The data will be anonymized and shared under request to the first author.

and contact information or URL).<br>• This text is appropriate if the data are owned by a third party and authors do not have permission to share the data.<br><br>* typeset	
Additional data availability information:	

Powered by Editorial Manager® and ProduXion Manager® from Aries Systems Corporation

Manuscript
Click here to access/download;Manuscript;Manuscript.docx

Title Page

Development and validation of scales for EFL speaking self-efficacy: Construct, sources, and relations

Yawen Wang; Peijian Paul Sun*
Corresponding author*
Peijian Paul Sun, Department of Linguistics, Zhejiang University, Hangzhou, China, 310058; Phone: +8619883177109; Email: luapnus@zju.edu.cn

ORCID iD
Yawen Wang https://orcid.org/0000-0002-3910-4865
Peijian Paul Sun https://orcid.org/0000-0003-4820-2915

Conflict of interest: The authors declare that there is no conflict of interest regarding the publication of this paper.

Ethical Statement: The study was approved by the Human Participants Ethics Committee of School of International Studies at Zhejiang University (SIS2022-01).

Informed Consent: Informed consent was obtained from all the participants involved in the study including their consent for paper publication based on the collected data.

Acknowledgement: The work was supported by the National Social Science Foundation of China (Grant no. 19CYY008).

Author Biography

Yawen Wang is a PhD Candidate at the Department of Linguistics, Zhejiang University, China. Her research interests include second language acquisition and quantitative linguistics. She has published papers in *System*, *Frontiers in Psychology*, and *Glottometrics*. Email: mileywyw@zju.edu.cn

Peijian Paul Sun (PhD, University of Auckland) is an associate professor at the Department of Linguistics, Zhejiang University, China. His research focuses on L2 speaking, L2 teaching, and educational technology. His publications have appeared in journals, such as *Applied Linguistics*, *TESOL Quarterly*, *Computer Assisted Language Learning*, and *System*, among others. He also serves as a guest associate editor for *Frontiers in Psychology*. Email: luapnus@zju.edu.cn

Development and validation of scales for EFL speaking self-efficacy: Construct, sources, and relations

Abstract

Speaking is not a compulsory language skill assessed in the English subject of the National College Entrance Examination in China. This explains why, in elementary and secondary schools, less focus has been placed on the development of English-speaking abilities among Chinese students, resulting in their unbalanced mastery of language skills. Although self-efficacy is a crucial factor influencing students' language performance, our understanding of speaking self-efficacy is insufficient in terms of its construct, its sources, and the relationships between the two. We, therefore, constructed psychometrically sound instruments to measure EFL speaking self-efficacy, including the EFL Speaking Self-Efficacy Scale (EFL-SSES) and the EFL Sources of Speaking Self-Efficacy Scale (EFL-SSSES), in accordance with Bandura's [1] self-efficacy theory. Additionally, we performed path analysis to understand the relationship between the construct and the sources of EFL speaking self-efficacy. The results revealed the key role of physiological and emotional states and marginal importance of vicarious experience for speaking self-efficacy, advancing our grasp of self-efficacy theory in the speaking domain. Our research sheds light on how to assist researchers and educators in identifying and enhancing students' speaking self-efficacy via a variety of sources.

Keywords: speaking self-efficacy, factor analysis, scale development, scale validation

1 Introduction

In China, English as a foreign language (EFL) learners' listening and speaking skills are often weaker than their writing and reading skills [2]. This phenomenon is mainly ascribed to the follow-up two aspects. Firstly, the monolingual context without an authentic English language environment cannot provide learners with the opportunities necessary for developing their English speaking competence. Secondly, pedagogical methods in middle and high schools in China have placed a low premium on speaking, resulting in learners' poor English-speaking abilities [3]. As a consequence, students may experience anxiety and vulnerability while expressing thoughts, conducting debates, or interacting with others during English-speaking assignments. Our primary interest in this study is finding ways to tackle these obstacles to English speaking. In this study, the English speaking construct refers to English learners' ability to express themselves in both monologic and interactive ways and to collaborate in the development and maintenance of interaction [4].

Since Bandura [5] firstly proposed the concept of self-efficacy, researchers have focused on determining how learners' self-efficacy may affect their learning process or outcomes [6,7]. Students that are more self-efficacious set higher objectives, put in more effort to finish academic assignments, and use a variety of flexible and diverse learning strategies. As Bandura [5] stated, concentrating on a specific context and activity domain would advance the understanding of self-efficacy. Speaking self-efficacy in this study is defined as the confidence in one's speaking ability to properly perform grammar, usage, communication, and interaction.

While numerous measures of EFL learners' self-efficacy have been developed [8], research related to self-efficacy in the domain of speaking remains scarce. Furthermore, Bandura [1] proposed that people acquire self-efficacy by interpreting information from four distinct sources. However, there is a dearth of research on sources of speaking self-efficacy among EFL learners. More attempt is in urgent need to elucidate the construct and sources of speaking self-efficacy and build robust instruments to deepen our knowledge of speaking self-efficacy. To bridge the gap, we developed and validated scales for EFL speaking self-efficacy in an attempt to answer the following three research questions (RQs).

RQ1: What is the construct of EFL speaking self-efficacy?

RQ2: What are the sources of EFL speaking self-efficacy?

RQ3: What is the relationship between the construct and the sources of EFL speaking self-efficacy?

2 Literature review

2.1 Theoretical construct of self-efficacy: Social-cognitive theory

Derived from the social cognition theory, self-efficacy refers to a person's judgment of his or her capabilities to complete a specific task with his or her skills [5,9]. As a personal factor in the form of beliefs, it has a major influence on how individuals approach goals, initiatives, and obstacles [10,11]. Students who have a higher level of self-efficacy tend to show positive orientations toward academic tasks. They are eager

to solve new issues and willing to persevere in the face of adversity. Consequently, their self-efficacy can be largely reinforced and improved. In contrast, people with poor self-efficacy constantly doubt their abilities to perform tasks and often avoid dealing with difficulties and challenges.

As contended by several researchers [e.g., 12], individuals' perceived self-efficacy may be contingent on the context, implying that the characteristics of specific tasks or situations even in one particular domain may influence their self-efficacy. Domain-specificity is a critical feature of self-efficacy [13], indicating that individuals' self-efficacy levels vary across different domains of activity. It is determined based on individuals' perceived competence to accomplish specific tasks in particular situations [14]. For instance, an EFL learner may be quite confident in his or her writing abilities, but may have poor self-efficacy in speaking. Thus, it is indispensable to explore self-efficacy by concentrating on a specific context and activity domain and developing questionnaires accordingly.

Studies on students' self-efficacy have received continual interest in the field of language acquisition [15,16]. Self-efficacy is a significant predictor of academic achievement in general [6,7,17] and language proficiency in particular [18,19]. Recently, Wang and Sun [20] resorted to meta-analysis to confirm a positive relationship between self-efficacy beliefs and language learning outcomes. This comprehensive review suggested that research regarding self-efficacy is thriving across diverse domains (overall, receptive and productive language skills), demonstrating a domain-specific feature of self-efficacy. This meta-analysis indicated that few studies

examined self-efficacy in listening and speaking compared to other domains, which may be accounted for by the fact that schools place a premium on students' literacy development, whilst neglect listening and speaking [21]. The research on how students' speaking self-efficacy functions in their learning process may still be insufficient and may warrant further investigation.

2.2 Construct of speaking self-efficacy

According to Bandura [5], researchers would find the most utility from self-efficacy by focusing on a specific context and activity domain. In other words, self-efficacy may be regarded as a domain-specific and multi-dimensional construct [12]. Theoretically, how individuals develop their self-efficacy is based on precise appraisals of specific actions or objectives that may be accomplished successfully in one particular domain [12,22].

Research on self-efficacy has lately shifted away from the context of English as the first language [14,17] toward the EFL context (15,23–25). Previous studies have noted that EFL students exhibited moderate levels of self-efficacy beliefs [26,27] and found that this characteristic is beneficial to language learning [28]. Numerous empirical investigations have further linked EFL learners' self-efficacy to their language skills, corroborating a more thorough and situational view of self-perceived competence [22]. Self-efficacy was found to be predictive of listening [29], reading [30], and writing [23,31,32]. As some researchers have suggested [14,33], studies of students' self-efficacy should avoid using general self-efficacy items and instead build more context-specific measures. In other words, students' speaking self-efficacy should

not be merely regarded as one unified measurement but as a collection of factors that may be analyzed further.

While numerous self-efficacy instruments exist, the vast majority of them focus on writing self-efficacy [18,20]. Only a few studies have attempted to develop questionnaires to assess students' speaking self-efficacy [34–36]. For example, Harris [34] developed the Communicative Self-Efficacy Questionnaire and found low to moderate correlations between learners' speaking and listening self-efficacy and their language proficiency. However, Sundari and Dasmo [35] found no evidence of the relationship between speaking self-efficacy and speaking behaviors, in contrast to previous research. The underlying reason is the lack of validity and reliability evidence for the self-efficacy construct measures used. Zhang et al. [36] focused on self-efficacy in English public speaking. Its results indicate that there barely existed any direct or indirect effect on English public speaking performance via English language proficiency and self-efficacy sources, though the four theoretical sources had different effects on self-efficacy. The above inconsistent findings suggest that the construct of speaking self-efficacy is promising for further investigation. Given that there is limited understanding of EFL learners' speaking self-efficacy, it is imperative to design and develop a robust scale for measuring EFL learners' speaking self-efficacy.

2.3 Self-efficacy measures

There are few studies designed to construct valid and reliable instruments for measuring self-efficacy. The most commonly used approach is self-report questionnaires. The Motivated Strategies for Learning Questionnaire (MSLQ) is the

most frequently used instrument [42]. The 8-item "Self-Efficacy for Learning and Performance" is one of the 15 subscales. Eight questions were developed to ascertain students' perceptions of their ability to succeed in an undergraduate course. Since its release, MSLQ has been widely examined for its psychometric qualities [e.g., 42,43].

In addition to instruments that conceptualize self-efficacy from a broad perspective, Wang [8] developed the Questionnaire of English Self-Efficacy (QESE) with 32 items in his dissertation to measure EFL learners' self-efficacy beliefs. Based on Bandura's [1] guidelines, the QESE met the demand for validity and reliability to probe EFL learners' self-efficacy beliefs. Each item demands students to judge their abilities to complete specific tasks in English listening, speaking, reading, and writing. The QESE is the most extensively used self-efficacy scale in the EFL field [15,16,44].

Apart from the QESE, Harris [34] recently developed the Communicative Self-Efficacy Questionnaire self-efficacy (SE) to measuring Japanese EFL learners' communicative self-efficacy concerning their speaking and listening skills with two potential components (speaking self-efficacy and listening self-efficacy).

In summary, due to the mix use of general and task-specific self-efficacy in EFL research, the findings of self-efficacy display inconsistency. There is a paucity of instruments to measure speaking self-efficacy in EFL settings. Therefore, this study aims to fill this gap.

2.4 Sources of speaking self-efficacy

According to social cognitive theory, expectations of personal efficacy are based on four sources of self-efficacy: mastery experience (ME), vicarious experience (VE),

social persuasion (SP), physiological and emotional states (PES) [5]. ME as the most influential factor of self-efficacy beliefs is the interpreted result of one's own previous accomplishments [9]. Success in overcoming difficult challenges boosts a person's self-efficacy, while repeated failures erode it. Interpretation of the outcomes of their efforts may have long-lasting effects on their self-efficacy [1,37].

Observing others' performances, known as VE, has a remarkable impact on self-efficacy [5]. Individuals evaluate their own chances of success or failure based on their observations of others who excel at or struggle with the same or comparable tasks. Successful actions of others often boost one's self-efficacy, and vice versa. It is worth mentioning that the classroom climate impacted greatly on students' vicarious experience [38]. In the competitive classroom, students' self-efficacy decreased when they engaged in vicarious learning, while students' self-efficacy did not show any significant change in the non-competitive classroom.

The third source of self-efficacy is SP [5]. Encouragement and positive feedback would strengthen one's self-efficacy, whilst discouragement and negative feedback would deteriorate it. Rather than directly generating self-efficacy beliefs, social persuasion assists in the development of self-efficacy when combined with other strategies [1,9]. Its impact is intimately attributed to the credibility and reputation of the information source. As previous research suggested, it may be easier to undermine an individual's self-efficacy via social persuasions than to enhance it [39]. Furthermore, social persuasion has a more substantial effect on learners' self-efficacy in collectivist cultures than in individualist cultures [10,40].

Last but not least, PES have a significant influence on the development of self-efficacy [5]. How individuals perceive their emotional reactions is of prime importance to their perceptions of themselves [1]. If a speaker lives in dread in anticipation of his or her speaking performance, he or she is likely to take it as evidence of inadequate speaking ability. Increasing individuals' physiological and emotional well-being and reducing negative emotional states may strengthen self-efficacy.

Bandura's [1,5] theoretical model of four sources of self-efficacy has been employed in a plethora of research to demonstrate its latent structure and predictive roles in different academic domains [11]. However, few instruments have been developed particularly to assess EFL learners' sources of speaking self-efficacy. Inadequate research has been conducted to ascertain the relationships between speaking self-efficacy and its sources among EFL learners. Only Zhang et al. [41] have investigated the self-efficacy of English public speaking and its sources. Given that the four sources serve as critical antecedents of speaking self-efficacy, there exists the need to further examine their relationships. Our present study serves as an initial step to develop and preliminarily validate scales for EFL speaking self-efficacy with an attempt to understand its construct, its sources, and the relationship between them.

3 Materials and methods

Informed by the social-cognitive theory [1], we sought to conceptualize and validate the multidimensional structures of EFL speaking self-efficacy and its sources. The whole study was carried out from 11th December 2022 to 30th January 2023. In Study 1, we developed and validated a quantitative instrument exploring college EFL

learners' speaking self-efficacy, namely EFL Speaking Self-Efficacy Scale (EFL-SSES). It went through three phases: (a) item generating, (b) exploratory factor analysis (EFA), and (c) confirmatory factor analysis (CFA). In Study 2, we developed and validated another instrument evaluating students' sources of speaking self-efficacy, namely EFL Sources of Speaking Self-Efficacy Scale (EFL-SSSES), based on the four sources proposed by Bandura [5]. It also went through the same three phases. In Study 3, we examined the relationships between speaking self-efficacy and its sources with path analysis. Please note that before data collection, online written consent forms were obtained from participants for their voluntary participation.

3.1 Study 1: EFL-SSES development and validation

3.1.1 Phase 1: Item generation

The process of item generation began with individual interviews. Ten undergraduate students were invited to voluntarily participate in the semi-structured interview with different gender, year level, and disciplinary major. S1 Appendix presents the outline of semi-structured interview, which was designed based on previous literature [8,42]. The analysis of the interview data helped to generate the initial items. The initial item pool included 30 speaking self-efficacy items. Since the original items were in Chinese, a method of forward-backward translations was used by both authors to ensure the conceptual equivalency of the English and Chinese language versions.

Two professors in the field of second language acquisition (SLA) were invited to

scrutinize the content validity of the initial item pool. These confusing and redundant items were reworded or eliminated based on their comments. The examination resulted in the deletion of 8 items, leaving 22 items for final analysis. The first version of the questionnaire was pilot-tested with 40 participants who had taken courses related to oral English. This led to the reformulation of some items for clarity and readability, eliminating three irrelevant and double-barreled items and rewording two others. This resulted in the formation of a preliminary 19-item scale with a 7-point Likert scale ranging from "1 = strongly disagree" to "7 = strongly agree".

3.1.2 Phase 2: EFA of EFL-SSES

Participants

A total number of 242 undergraduate students from one southeast university in China voluntarily participated in this phase. They are non-English major students with Mandarin Chinese as their native language. They were recruited based on convenience sampling. At the time when they completed the questionnaire, they had taken their English courses for at least one semester and had prior experiences delivering oral presentations in English. Participants were briefed on the purpose of the study and were requested to respond honestly and sincerely to the items. They spent on average 5 minutes completing the randomized Chinese version of the EFL-SSES. Fifteen questionnaires were discarded as the participants did not complete them, leaving 227 valid questionnaires. The response rate was 93.80%. Their average age was 19 years old (M = 18.94, SD = 0.85), with 43% female (N = 97) and 57% male (N = 130). They

reported an average 11 years of formal English language learning (*M* = 10.75, *SD* = 1.76). Participants came from four majors: Engineering (38%, *N* = 87), Liberal arts (29%, *N* = 65), Science (20%, *N* = 45), and Medicine (13%, *N* = 30).

Results

EFA was conducted to test the hypothesized factor structure and refine the items through SPSS version 24 [45]. The mean values of all the items ranged from 3.21 to 4.67. The standard deviations ranged from 0.08 to 0.10, with the skew and kurtosis indices from -0.84 to 0.57 and -0.51 to 0.37 respectively. The data in the present study were considered to be univariate normal. The results of the Kaiser–Meyer–Olkin test verified the sample adequacy (KMO = 0.91). The result of Barlett's test of sphericity (χ^2 (105) = 2595.97, $p < 0.001$) indicated that correlations between items were appropriate for factor analysis.

To determine the underlying structure of EFL-SSES, the 19 items were subjected to EFA with maximum likelihood analysis and a promax rotation method. Four factors with eigenvalues above 1.0 were extracted. The EFA was repeated until all items in each factor had reasonably high loadings (greater than 0.4) and no more items could be excluded from the analysis. According to the pattern matrix from EFA and the internal consistency reliability results, 15 of the 19 original items in the EFL-SSES loaded high on the four factors, as confirmed by the scree plot with parallel analysis.

Insert Fig 1 around here.

Fig 1. The scree plot with parallel analysis of EFL-SSES

These factors were labelled linguistic self-efficacy (LSE), self-regulatory efficacy

(SRE), delivery self-efficacy (DSE), and performance self-efficacy (PSE) based on their contents. The four factors accounted for 75.69 % of the variance. The Cronbach's alpha (α) was used to check the internal consistency reliability of each extracted factor. Co-efficiencies of all four factors exceeded the threshold of 0.7 (see Table 1). S2 Appendix presents the finalized items of EFL-SSES. LSE refers to students' judgements of their capability to execute various phonological, lexical, syntax, and discourse skills necessary to communicate effectively in English in the classroom. SRE is associated with EFL learners' perceived capability to execute metacognitive strategies while speaking English in class. DSE refers to EFL learners' speaking ability to talk with emotional appeal by using self-control in the classroom. PSE represents students' perceptions of their speaking capability to accomplish course assignments or comprehension of course material.

Table 1. Results of EFA and reliabilities of the four-factor EFL-SSES

Factor	Items	Factor loadings	Total Variance Explained	Cronbach's alpha (α)
LSE	5	0.549-0.895	7.78%	0.884
SRE	3	0.414-0.871	5.45%	0.780
DSE	3	0.564-0.980	5.56%	0.907
PSE	4	0.627-0.932	56.90%	0.893

3.1.3 Phase 3: CFA of EFL-SSES

After administering the EFA analysis, CFA was conducted to determine the factorial validity of the 15-item EFL-SSES via SPSS [45] and AMOS 24 software [46]. The model fit is evaluated based on multiple fit indices, including χ^2 significance test, Comparative Fit Index (CFI), the Goodness of Fit Index (GFI), the Tucker–Lewis Index (TLI), the Standardized Root Mean Square Residual (SRMR), and the Root Mean Square Error of Approximation (RMSEA). For a model with a sample size larger than 250 and its observed variable number greater than 30, CFI, TLI, and GFI above 0.92, SRMR below 0.08, and RMSEA below 0.07 indicate adequate fit [47]. The reliability of the scale was examined by Cronbach's alpha (α). Average variance extracted (AVE) and composite reliability (CR) were used to test the convergent validity of the scale. The convergent validity of the construct is acceptable if AVE is larger than 0.5 and CR is higher than 0.6 [48]. The heterotrait-monotrait ratio of correlations (HTMT) proposed by Henseler et al. [49] was used to test the divergent validity. If the HTMT score is less than 0.90, discriminant validity between two reflective notions has been established.

Participants

To conduct CFA, the scale was administered to 300 non-English major undergraduate students with Mandarin Chinese as their native language. They did not take part in the previous EFA phase and were recruited based on convenience sampling. Participants spent on average 5 minutes completing the randomized Chinese version of the EFL-SSES. The questionnaires from 11 students were disregarded because of missing values and inconsistent responses to care-check items. This reduced the number of participants to 289. The response rate was 96.33%. They consisted of 170 males

(59%) and 119 females (41%). Their age ranged from 17 to 21 with an average of 18.80 (*SD* = 0.68). They reported an average 11 years of formal English language learning (*M* = 10.79, *SD* = 1.88). All participants were from four majors: Engineering (37%, *N* = 107), Science (30%, *N* = 86), Liberal arts (23%, *N* = 66), and Medicine (10%, *N* = 30).

Results

CFA was conducted to validate the underlying factor structure. The mean score of all 15 items ranged from 3.50 to 5.18, with standard deviations ranging from 1.12 to 1.41. The data were univariate normal, with skewness ranging from -0.84 to 0.30 and kurtosis values ranging from -0.52 to 0.90. Based on the theoretical understanding of self-efficacy, a zero-order correlated model was proposed (Model 1). Maximum likelihood estimation was used to estimate the model. This model assumed that the underlying four factors of speaking self-efficacy were theoretically correlated with each other. It is supported by the fact that participants can distinguish the four dimensions of self-efficacy, but the four factors are intercorrelated with one another. The CFA analysis demonstrated a reasonable fit to the data, $\chi^2(84) = 199.785$, $p < .001$, CFI = 0.963, GFI = 0.918, TLI = 0.954, SRMR = 0.0405, and RMSEA = 0.069 [0.057~0.082]. Figure 2 showed the standardized regression weights of Model 1. The items were all significantly related to their underlying constructs and the factor loadings ranged from 0.513 to 0.815. Cronbach's alpha coefficient indicated a robust internal reliability of the scale. The results in Table 2 prove the convergent and discriminant validity of EFL-SSES.

Table 2. Results of convergent and discriminant validity of the four-factor EFL-SSES

Factor	HTMT				Cronbach's alpha (α)	CR	AVE
	1	2	3	4			
LSE					0.89	0.83	0.51
SRE	0.65				0.85	0.86	0.67
DSE	0.83	0.75			0.90	0.78	0.56
PSE	0.80	0.68	0.86		0.88	0.83	0.57

One of the advantages with the CFA is that it enables the comparison of alternative conceptualizations of the underlying factor structure of the instrument (Noar, 2003). As a result, two other models were proposed. Model 2 hypothesized a one-factor model in which all the items loaded on one factor, since participants perceived all these items of speaking self-efficacy as a unidimensional construct. According to Model 3, the four components of speaking self-efficacy were uncorrelated. It suggests that LSE, SRE, DSE, and PSE are unrelated constructs. The model fit indices of the three models were compared (Table 3). The comparison results revealed that Model 1 had the best fit indices when compared to Model 2 and Model 3, providing support to the construct validity of the four-dimension correlated structure of speaking self-efficacy (Model 1).

Insert Fig 2 around here.

Fig 2. Four-factor correlated model of EFL speaking self-efficacy.

Table 3. Goodness-of-Fit Indices for Competing Models of EFL-SSES

Model	ML χ^2	*df*	χ^2/df	CFI	GFI	TLI	SRMR	RMSEA	RMSEA 90% CI
Model 1	199.785	84	2.378	0.963	0.918	0.954	0.0405	0.069	0.057-0.082
Model 2	617.174	90	6.857	0.833	0.75	0.805	0.0695	0.143	0.132-0.153
Model 3	879.375	90	9.771	0.749	0.697	0.708	0.4296	0.175	0.164-0.185

3.2 Study 2: EFL-SSSES development and validation

3.2.1 Phase 1: Item generation

The process of item generation was guided by Bandura's theoretical work, which proposes the four sources of self-efficacy [5]. After examining all the available literature on sources of self-efficacy [11,41], the initial 30 items were constructed. The questionnaire items were reworded to accommodate EFL learners' speaking learning process. The English version scale presented was then translated into Chinese. In order to prevent potential loss of meaning, the first and second authors as native Mandarin Chinese and proficient English bilingual speakers carefully examined the items and their Chinese translations back and forth.

Two university professors with expertise in SLA and extensive experience teaching EFL speaking were invited to review the initial pool of items for relevance, clarity, redundancy and readability. Based on their suggestions, 10 items that were

deemed confusing, irrelevant, and difficult to comprehend were eliminated, leaving 20 items for the final analysis. It was pilot-tested with 35 students from a Chinese university who had attended oral English courses. Following their feedback, four items were deleted. The final questionnaire consists of 16 items with a 7-point Likert scale.

3.2.2 Phase 2: EFA of EFL-SSSES

Participants

230 students were invited to respond to the scale voluntarily. This was a convenience sample. They were non-English major undergraduate students with Mandarin Chinese as their native language. Participants spent on average 5 minutes completing the randomized Chinese version of the SSSER. Six students' responses were disregarded because of missing values and inconsistent answers to care-check items. This reduced the number of participants to 224, with 138 males (62%) and 86 females (38%). The response rate was 97.39%. They ranged in age from 16 to 21 years with a mean of 18.8 (*SD* = 0.72). They reported an average of 11 years' experience studying English (*M* = 10.74, *SD* = 1.90). Participants came from four majors: Science (33%, *N* = 74), Engineering (32%, *N* = 72), Liberal arts (21%, *N* = 48), and Medicine (13%, *N* = 30).

Results

EFA was carried out as Study 1. All items have the mean values from 3.53 to 5.21. The standard deviations varied from 1.22 to 1.50. The skewness and kurtosis indices

varied from -0.90 to 0.33 and -0.55 to 1.00 respectively. The data were univariate normal. The KMO test confirmed the sample adequacy (KMO = 0.84). The result of Barlett's test of sphericity (χ^2 (78) = 2703.87, p < .001) indicated that correlations between items were suitable for factor analysis. EFA with maximum likelihood estimation and a promax rotation method was performed. We sought to develop the scale with concise sets of indicators and thereby only retained items with high loadings of 0.40 or above from the initial pool of EFL-SSSES. The EFA based on the 16 items yielded a four-factor solution, as confirmed by Figure 3.

Insert Fig 3 around here.

Fig 3. The scree plot with parallel analysis of EFL-SSSES.

Remaining items loaded adequately on their respective factors. Following that, we eliminated three redundant items with lower loadings on the grounds of parsimony. This step resulted in a more succinct solution, since the remaining 13 items adequately covered Bandura's [5] sources of self-efficacy (see details in Table 4). These items account for 85.77% of the variance in total. Cronbach's alpha coefficient of internal consistency of the subcategories was much higher than 0.70. S3 Appendix presents the finalized items of EFL-SSSES.

Table 4. Results of EFA and reliabilities of the four-factor EFL-SSSES

Factor	Items	Factor loadings	Total Variance Explained	Cronbach's alpha (α)
ME	4	0.695-1.020	48.31%	0.926
VE	3	0.804-1.033	17.80%	0.930

SP	3	0.806-0.894	8.56%	0.911
PES	3	0.817-0.999	11.10%	0.920

3.2.3 Phase 3: CFA of EFL-SSSES

Participants

CFA was implemented through administering the scale generated in the EFA to 307 non-English major undergraduate students whose native language is Mandarin Chinese. They were selected based on convenience sampling and did not participate in the previous EFA study. Participants spent on average 5 minutes completing the Chinese version of the EFL-SSSES. Questionnaires from 12 students with missing values and inconsistent responses were omitted, which reduced the number of participants to 295 (169 males and 126 females). The response rate was 96.09%. Their age ranged from 17 to 23 with a mean of 18.91 (*SD* = 0.88). They reported an average 11 years of formal English language learning (*M* = 10.69, *SD* = 1.90). All participants were from four majors: Science (38%, *N* = 111), Liberal arts (27%, *N* = 80), Engineering (22%, *N* = 66), and Medicine (13%, *N* = 38).

Results

CFA was implemented to validate the underlying factors using maximum likelihood estimation. The four latent factors were assumed to be correlated and allowed to covary in the model. The mean of all items was between 3.72 and 5.87. Standard deviations varied between 1.04 and 1.50, whereas skew and kurtosis indices ranged

from -1.16 to 0.15 and -0.81 to 2.20, respectively. The purpose of this study was to test the four-factor correlated model of speaking self-efficacy sources (Model 4). Each of the indices revealed that the data fitted the model rather well ($\chi^2(59) = 182.828$, $p < .001$, CFI = 0.957, GFI = 0.912, TLI = 0.943, SRMR = 0.0554, and RMSEA = 0.084 [0.071~0.099]). The standardized regression weights of the four-factor correlated model of sources of speaking self-efficacy are shown in Figure 4. Each item was significantly related to its underlying construct, with factor loadings ranging from 0.628 to 0.925. Cronbach's alpha coefficients indicated that the scale has a robust internal reliability. Table 5 demonstrates the convergent and discriminant validity of the EFL-SSSES.

This study compared the four-factor correlated model of sources of speaking self-efficacy (Model 4) to a null model (Model 5) and an uncorrelated four-factor model (Model 6). In Model 5, we hypothesized that all the 13 items loaded on one overarching factor. This model is supported by the fact that participants perceived all these sources of speaking self-efficacy as a unidimensional construct. Model 6 included four uncorrelated factors representing sources of speaking self-efficacy. A support for this model suggests that mastery experience, vicarious experience, social persuasion, and physiological and emotional states are unrelated constructs. The fit indices for three models were provided in Table 6. The comparison findings revealed that Model 4 had the best fit indices when compared with Model 5 and Model 6, ensuring the construct validity of the four-dimension correlated structure of sources of speaking self-efficacy (Model 4).

Insert Fig 4 around here.

Fig 4. Four-factor correlated model of sources of EFL speaking self-efficacy.

Table 5. Results of convergent and discriminant validity of the four-factor EFL-SSSES

Factor	HTMT				Cronbach's alpha (α)	CR	AVE
	1	2	3	4			
ME					0.91	0.89	0.67
VE	0.57				0.84	0.85	0.66
SP	0.72	0.50			0.91	0.91	0.76
PES	-0.34	0.02	-0.27		0.90	0.91	0.78

Table 6. Goodness-of-Fit Indices for Competing Models of EFL-SSSES

Model	ML χ^2	*df*	χ^2/df	CFI	GFI	TLI	SRMR	RMSEA	RMSEA 90% CI
Model 4	182.828	59	3.099	0.957	0.912	0.943	0.0554	0.084	0.071-0.099
Model 5	1371.852	65	21.105	0.548	0.578	0.457	0.1642	0.262	0.250-0.274
Model 6	472.090	65	7.263	0.859	0.798	0.831	0.2934	0.146	0.134-0.158

3.3 Study 3: Relationships between EFL-SSES and EFL-SSSES

Responses from students that participated in both Study 1 and Study 2 went through path analysis to verify the relationship between speaking self-efficacy and its sources.

3.3.1 Participants

There were 304 students that participated in both Study 1 and Study 2. Their average age was 19 years old (*M* = 18.87, *SD* = 0.79), with 61% male (*N* = 184) and 39% female (*N* = 120). They reported having studied English for an average of 11 years (*M* = 10.75, *SD* = 1.90). They came from four majors: Engineering (34%, *N* = 103), Science (27%, *N* = 82), Liberal arts (26%, *N* = 80), and Medicine (13%, *N* = 39).

3.3.2 Results

Path analysis was conducted to ascertain the predictive power of each underlying source in the four aspects of speaking self-efficacy. The goodness of fit indices were indicative of an acceptable model-to-data fit ($\chi^2(333) = 896.755$, $p < .001$, CFI = 0.920, GFI = 0.821, TLI = 0.909, SRMR = 0.1735, and RMSEA = 0.075 [0.069~0.081]).

Insert Fig 4 around here.

Fig 5. Path analysis and path coefficients of EFL speaking self-efficacy and its sources.

As Figure 5 illustrates, mastery experience is the most significant source of EFL learners' speaking self-efficacy and has the greatest explanatory power for enhancing students' confidence in their abilities to deliver fluent oral presentations (LSE), regulate their metacognitive behavior (SRE), maintain emotional control (DSE), and manage their speaking performance (PSE). Notably, physiological and emotional states is the second most significant predictors of EFL learners' speaking self-efficacy in the four EFL-SSES aspects. It has significantly negative contributions to LSE, SRE, DSE, and PSE. This demonstrates weak physiological and mental states may undermine EFL learners' confidence in their speaking ability. Nonetheless, social persuasion is a

substantial and positive predictor of DSE and PSE. These results suggest that positive feedback from others may boost EFL learners' confidence in delivering speaking performances during English courses. Vicarious experience is a marginally significant predictor of PSE. In other words, modeling has a limited influence on EFL learners' confidence in speaking English.

4. General discussion

The purpose of this investigation is to develop and evaluate speaking self-efficacy instruments for EFL learners. These instruments provide a new lens through which to view EFL learners' speaking efficacy and its sources. It is a first attempt to capture the construct of speaking self-efficacy into the development of speaking ability. The items were generated in accordance with the self-efficacy theory and early research on speaking skill. The newly developed EFL-SSES and EFL-SSSES instruments was validated by EFA and CFA, with satisfactory validity and reliability of their factor structure. Notably, the final EFL-SSES is composed of 15 items concerning speaking self-efficacy. Bandura's [5] four sources of self-efficacy are reflected in the final EFL-SSSES by 13 items. EFA and CFA provided significant support for the factorial structure of EFL-SSES and EFL-SSSES. The validation results of these instruments suggest that the four sources could serve as major predictors of the EFL learners' speaking self-efficacy. The path analysis results revealed that four sources of EFL learners' speaking self-efficacy played significant roles in their speaking self-efficacy. In general, the findings not only validated the EFL-SSES and EFL-SSSES psychometrically sound instruments of speaking self-efficacy and its sources, but also

demonstrated that their factor structures could be reliably distinguished on both conceptual and empirical grounds.

4.1 Multiple dimensions of EFL-SSES and EFL-SSSES

The EFL-SSES and EFL-SSSES were created with the primary goal to develop psychometrically sound instruments of speaking self-efficacy. With 15 items on the finalized EFL-SSES, EFA yielded a four-factor structure. This structure was supported by CFA results. LSE refers to an EFL learner's capacity to communicate in a clear, logical, and coherent manner. Given the positive relationship between self-efficacy and English language proficiency, we hypothesize that learners with high scores would be more self-efficacious in their learning process. SRE indicates EFL learners' confidence in their ability to control their metacognitive behavior. According to prior research, students' confidence in self-regulated learning is a significant predictor of academic achievement [11]. We believe that the high-scoring students would speak English frequently. DSE reflects students' overall speaking performance in the classroom, including their use of emotional appeal and self-control. As expected, high-scoring students would perform better in their speaking assignments. PSE discusses how individuals' perceptions of their abilities affect their behavior in speaking classes. Specifically, the higher-scoring students would conduct better in the classroom.

EFA and CFA on the final 13-item EFL-SSSES produced a four-factor solution, in line with the self-efficacy theory [1]. ME refers to cognitive interpretations of prior performance achievements. It has been regarded as the most reliable and substantial source of personal efficacy information [11]. We propose that EFL learners' confidence

may be boosted when they see their past learning experiences as constructive, while failures reduce their learning confidence. VE demonstrates that when EFL learners lack mastery experience, they obtain necessary knowledge about the performance of respected models in order to evaluate their own potential [39]. It is reasonable to believe that observing role models succeed can favorably reinforce individuals' confidence in performing comparable or identical assignments. SP refers to how EFL learners often depend on important individuals, such as parents, instructors, and peers, for evaluative feedback, judgements, and appraisals about their academic achievement [5]. We believe that when learners acquire efficacy information from others, they are implicitly primed to raise or decrease the effort put on a particular task. PES indicates that EFL learners would gauge their confidence by recognizing sources of emotional arousal in comparable learning situations. We speculate that EFL learners who are confident in their ability to successfully complete given tasks will experience positive emotions, whereas those who experience negative emotions will experience a decline in self-efficacy, impairing performance and resulting in unsatisfactory learning outcomes [11].

4.2 Relationship between the construct and the sources of speaking self-efficacy

The path analysis results demonstrated four sources of speaking self-efficacy are crucial, exerting a strong influence on EFL learners' varied aspects of speaking self-efficacy. According to Figure 5, mastery experience was the most influential source of speaking self-efficacy across the four dimensions of speaking self-efficacy, consistent with previous findings [11]. EFL learners' interpretations of mastery experiences have

an effect on their speaking self-efficacy for comprehending cognitive aspects of the language acquisition process. As predicted, the EFL learners' prior successful speaking experiences influenced their self-confidence in presenting oral English, adopting metacognitive strategies, delivering self-controlled speaking presentations, and appraising their speaking competence. Language educators and practitioners can employ instructional techniques, such as task-based language teaching, to promote the development of meaningful experiences and confidence in EFL learners. Then, students' speaking self-efficacy in the domain of mastery experience might be significantly increased.

Notably, the findings indicate that EFL learners' physiological and emotional states are the second most powerful predictors of their linguistic self-efficacy, self-regulatory efficacy, delivery self-efficacy, as well as performance self-efficacy. Since schools give priority to students' reading and writing ability development but neglect listening and speaking [21], students may be concerned about the discrepancy between their real speaking performance and what they learned in English textbooks. Many non-native English instructors in China continue to place a premium on the accuracy of vocabulary and grammar rules, as well as repeating dialogues and passages from textbooks [50]. Once students discover incongruence between their prior knowledge and their actual spoken English performance in class, they may have elevated feelings of anxiety, tension, and stress related with the classroom speaking activity. For less efficacious students, speaking in the input-poor environment may trigger negative emotional states and a feeling of incompetence. These inadequate emotional arousals may be indicative

of a lack of ability to accomplish spoken English assignments. In other words, perceived cognitive incongruence may influence students' judgment of their own capacities to give speaking performances, then weakening their confidence.

As findings indicate, favorable verbal and nonverbal evaluations from others (social persuasion) contribute to delivery self-efficacy and performance self-efficacy. The encouragement and positive comments received from others may improve EFL learners' engagement in exhibiting advanced cognitive abilities and thoughtfully expressing their viewpoints in a group discussion. In turn, their confidence in oral English would be bolstered in these aspects. The path analysis findings imply that social persuasion works in tandem with mastery experience and physiological and emotional states. It is in line with previous findings [1,11] that social persuasion is effective only when combined with other sources affecting self-efficacy.

The efficacy information gained from vicarious experience is not very crucial for EFL learners. It has just a marginal predictive power to self-regulatory efficacy and performance self-efficacy. Due to the prevalence of normative assessment in the classroom, the environment is very competitive. These academic contests and comparisons may also act as a benchmark against which learners may assess their self-efficacy. As Chan and Lam [38] previously noted, when students participate in a competitive classroom, their self-efficacy may be jeopardized. Consequently, it is unknown how such vicarious experiences from professors or classmates will affect students' capacities and probability of success. The results imply that educators may use constructivist teaching approaches to foster a constructive and collaborative

environment in which students collaborate with their classmates and instructors. Additional educational activities emphasizing social interactions and interpersonal connections may help students understand the significance of vicarious learning.

Even though some absent relationships in Figure 5 may seem to be reasonably present, the predictive effects may be diminished while considering the overall model estimated through path analysis instead of the SEM technique. This may imply that the relationships between the two multidimensional constructs are more complex than expected. Future research should be aware of how to thoughtfully formulate models and establish the latent structural relationships based on the findings of this study.

5. Implications and limitations

The investigation has educational significance. First, the results indicate that instructors should engage students in realistic English-speaking activities that foster speaking self-efficacy in four aspects. Teachers can design effective teaching methods to boost EFL learners' speaking self-efficacy, especially when considering the sources of self-efficacy. This may involve the use of constructivist teaching approaches and task-based language teaching. Additionally, teachers should be aware of students' perceptions of their speaking experiences and work with them to accumulate positive mastery experiences. Moreover, instructors should assist learners to relieve those negative emotional arousals, such as frustration, which occur naturally throughout the learning process. Finally, instructors should be attentive to how students interact with one another throughout the speaking activities. These potential interactions may give beneficial information for students to foster their speaking self-efficacy.

While the results provided preliminary support for the reliability and validity of the EFL-SSES and EFL-SSSES, some limitations must be addressed. For starters, since the data came from the convenience sampling, our results may not be representative of the entire Chinese EFL learners. We mainly focused on EFL learners who had a high degree of English proficiency. The four-factor models proposed here may be inapplicable to low-level EFL learners. Second, future research should focus on the subtle relationships between how learners understand information from the four sources with the aid of qualitative methods, such as classroom observations or semi-structured interviews. Moreover, incorporating a variety of possible stakeholders, such as administrators, instructors, and parents, would provide a more systematic view of EFL learners' speaking self-efficacy and its sources. To obtain further understanding, it would be beneficial for researchers to follow the progress of EFL learners' speaking self-efficacy and its sources using a longitudinal study methodology.

References

1. Bandura A. *Social foundations of thought and action: A social cognitive theory*. Englewood Cliffs, NJ: Prentice-Hall; 1986.
2. Wei R, Su J. Surveying the English language across China. World Englishes. 2015; 34(2):175–89.
3. Wei R, Su J. Zhongguo neidi waiyu shiyong qingkuang diaocha fenxi [An analysis of foreign language use in Mainland China]. *Zhongguo Shehui Yuyanxue [The Journal of Chinese Sociolinguistics]*. 2008; 2:9–24.
4. Liu L, Jia G. Looking beyond scores: validating a CEFR-based university speaking assessment in Mainland China. *Language Testing in Asia*. 2017; 7(1):2.
5. Bandura A. *Self-efficacy: The exercise of control*. New York, NY: Worth Publishers; 1997.
6. Komarraju M, Nadler D. Self-efficacy and academic achievement: Why do implicit beliefs, goals, and effort regulation matter? *Learning and Individual Differences*. 2013; 25:67–72.
7. Wilson K, Narayan A. Relationships among individual task self-efficacy, self-regulated learning strategy use and academic performance in a computer-supported collaborative learning environment. *Educational Psychology*. 2016; 36(2):236–53.
8. Wang C. *Self-regulated learning strategies and self-efficacy beliefs of children learning English as a second language* [Ph.D. thesis]. The Ohio State University; 2004.
9. Bandura A. Self-efficacy: Toward a unifying theory of behavioral change. *Psychological Review*. 1977; 84(2):191–215.
10. Klassen RM. A cross-cultural investigation of the efficacy beliefs of South Asian immigrant and Anglo Canadian nonimmigrant early adolescents. *Journal of Educational Psychology*. 2004; 96(4):731–42.
11. Usher EL, Pajares F. Sources of self-efficacy in school: Critical review of the literature and future directions. *Review of Educational Research*. 2008; 78(4):751–96.
12. Bong M, Skaalvik EM. Academic self-concept and self-efficacy: How different are they really? *Educational Psychology Review*. 2003; 15(1):1–40.
13. Bandura A. Guide for constructing self-efficacy scales. In: Urdan T, Pajares F, editors. *Self-efficacy beliefs of adolescents*. Greenwich: Information Age

Publishing; 2006. p. 307–37.
14. Pajares F. Self-efficacy beliefs in academic settings. *Review of Educational Research*. 1996; 66(4):543–78.
15. Bai B, Chao GCN, Wang C. The relationship between social support, self-efficacy, and English language learning achievement in Hong Kong. *TESOL Quarterly*. 2019; 53(1):208–21.
16. Woodrow L. College English writing affect: Self-efficacy and anxiety. *System*. 2011; 39(4):510–22.
17. Zimmerman BJ, Kitsantas A. Homework practices and academic achievement: The mediating role of self-efficacy and perceived responsibility beliefs. *Contemporary Educational Psychology*. 2005; 30(4):397–417.
18. Bruning R, Dempsey M, Kauffman DF, McKim C, Zumbrunn S. Examining dimensions of self-efficacy for writing. Journal of Educational Psychology. 2013; 105(1):25–38.
19. Sanders-Reio J, Alexander PA, Reio TG, Newman I. Do students' beliefs about writing relate to their writing self-efficacy, apprehension, and performance? *Learning and Instruction*. 2014;33:1–11.
20. Wang C, Sun T. Relationship between self-efficacy and language proficiency: A meta-analysis. *System*. 2020; 95:102366.
21. Ferris D, Tagg T. Academic listening/speaking tasks for ESL students: Problems, suggestions, and implications. *TESOL Quarterly*. 1996; 30(2):297–320.
22. Pintrich PR, Schunk DH. *Motivation in education: Theory, research, and applications*. 2nd edition. Upper Saddle River, N.J: Prentice Hall; 2002.
23. Chen J, Zhang LJ, Chen X. L2 learners' self-regulated learning strategies and self-efficacy for writing achievement: A latent profile analysis. *Language Teaching Research*. 2022;13621688221134968. https://doi.org/10.1177/13621688221134
24. Derakhshan A, Fathi J. Grit and foreign language enjoyment as predictors of EFL learners' online engagement: The mediating role of online learning self-efficacy. *The Asia-Pacific Education Research*. 2023. https://doi.org/10.1007/s40299-023-00745-x
25. Li C, Zhang LJ. The development of accuracy and fluency in second language (L2) speaking related to self-efficacy through online scaffolding: A latent growth curve modeling analysis. *Journal of Psycholinguistic Research*. 2023.

https://doi.org/10.1007/s10936-023-09950-7

26. Wang C, Hu J, Zhang G, Chang Y, Xu Y. Chinese college students' self regulated learning strategies and self-efficacy beliefs in learning English as a foreign language. *Journal of Research in Education*. 2012; 22(2):103–35.
27. Wang C, Schwab G, Fenn P, Chang M. Self-efficacy and self-regulated learning strategies for English language learners: comparison between Chinese and German college students. *Journal of Educational and Developmental Psychology*. 2013; 3(1):173-191.
28. Kim DH, Wang C, Ahn HS, Bong M. English language learners' self-efficacy profiles and relationship with self-regulated learning strategies. *Learning and Individual Differences*. 2015; 38:136–42.
29. Fathi J, Derakhshan A, Torabi S. The effect of listening strategy instruction on second language listening anxiety and self-efficacy of Iranian EFL learners. *Sage Open*. 2020; 10(2):2158244020933878.
30. Shang HF. Reading strategy use, self-efficacy and EFL reading comprehension. *Asian EFL Journal*. 2010; 12(2):18–42.
31. Fathi J, Derakhshan A. The effect of strategy-based instruction on writing self-efficacy and writing anxiety of Iranian EFL learners. *Language and Translation Studies*. 2019; 51(4):139–61.
32. Zhang J, Zhang LJ, Zhu Y. Development and validation of a genre-based second language (L2) writing self-efficacy scale. *Frontiers in Psychology*. 2023. https://doi.org/10.3389/fpsyg.2023.1181196
33. Multon KD, Brown SD, Lent RW. Relation of self-efficacy beliefs to academic outcomes: A meta-analytic investigation. *Journal of Counseling Psychology*. 1991; 38(1):30–8.
34. Harris J. Measuring listening and speaking self-efficacy in EFL contexts: The development of the Communicative SE Questionnaire. *Language Teaching Research*. 2022. https://doi.org/10.1177/136216882210916.
35. Sundari H, Dasmo D. The effect of speaking self-efficacy and gender in speaking activities. *Jurnal Pendidikan Bahasa dan Sastra*. 2014; 14(2):205–15.
36. Zhang X, Ardasheva Y, Austin BW. Self-efficacy and English public speaking performance: A mixed method approach. *English for Specific Purposes*. 2020; 59:1–16.

37. Schunk DH, Pajares F. Self-efficacy theory. In: Wentzel KR, Miele DB, editors. *Handbook of motivation at school*. New York, NY: Routledge; 2009. p. 35–53.
38. Chan JCY, Lam S fong. Effects of competition on students' self-efficacy in vicarious learning. *British Journal of Educational Psychology*. 2008; 78(1):95–108.
39. Usher EL, Pajares F. Sources of self-efficacy in mathematics: A validation study. *Contemporary Educational Psychology*. 2009;34(1):89–101.
40. Ahn HS, Usher EL, Butz A, Bong M. Cultural differences in the understanding of modelling and feedback as sources of self-efficacy information. *British Journal of Educational Psychology*. 2016; 86(1):112–36.
41. Zhang X, Ardasheva Y, Egbert J, Ullrich-French SC. Building assessments for self-efficacy in English public speaking in China. *The Asia-Pacific Education Researcher*. 2019; 28(5):411–20.
42. Pintrich PR, Smith DAF, Garcia T, Mckeachie WJ. Reliability and predictive validity of the Motivated Strategies for Learning Questionnaire (MSLQ). *Educational and Psychological Measurement*. 1993; 53(3):801–13.
43. Sachs J, Law Y kum, Chan CKK, Rao N. A nonparametric item analysis of the Motivated Strategies for Learning Questionnaire—Chinese version. *Psychologia*. 2001; 44(3):197–208.
44. Zhang YY, Guo H. A study of English writing and domain-specific motivation and self-efficacy of Chinese EFL learners. Journal of Pan-Pacific Association of *Applied Linguistics*. 2012; 16(2):101–21.
45. IBM. *IBM SPSS statistics 24 core system user's guide*. USA: IBM Corporation.; 2016.
46. Arbuckle JL. *Amos 24.0 user's guide*. Chicago: IBM SPSS; 2016.
47. Hair JF, Black WC, Babin BJ, Anderson RE. *Multivariate data analysis*. 8th ed. Andover, Hampshire, UK: Cengage; 2019.
48. Fornell C, Larcker DF. Evaluating structural equation models with unobservable variables and measurement error. *Journal of Marketing Research*. 1981; 18(1):39–50.
49. Henseler J, Ringle CM, Sarstedt M. A new criterion for assessing discriminant validity in variance-based structural equation modeling. *Journal of the Academy of Marketing Science*. 2015; 43(1):115–35.
50. Hu G, Li L, Lei J. English-medium instruction at a Chinese University: Rhetoric

and reality. *Language Policy*. 2014; 13(1):21–40.

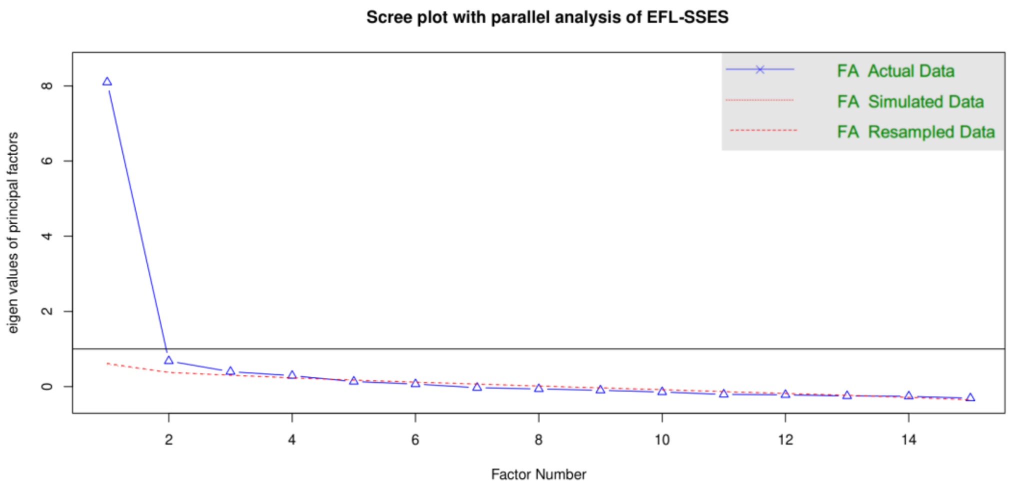

Fig 1. The scree plot with parallel analysis of EFL-SSES

Note. The point where the simulated eigenvalue (the red lines) meets the corresponding, real eigenvalue (the blue line) suggests the number of factors.

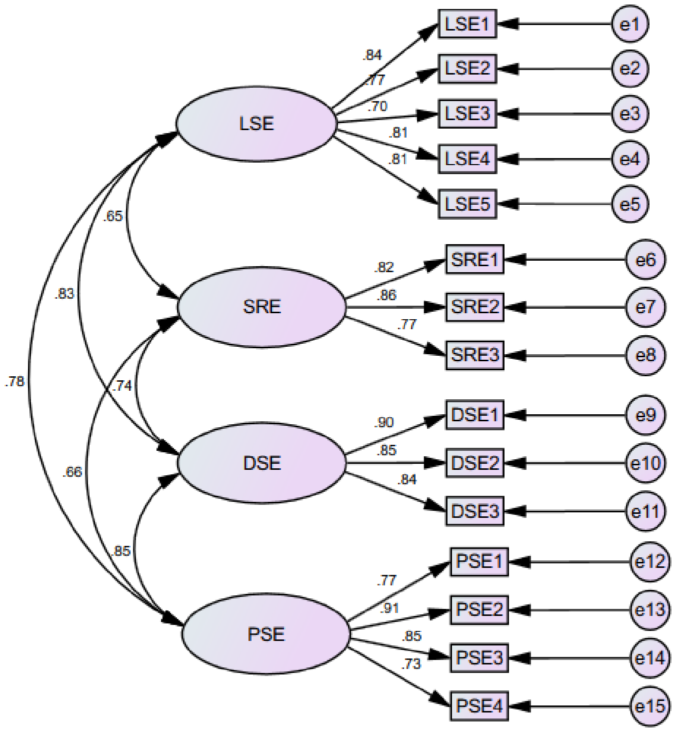

Fig 2. Four-factor correlated model of EFL speaking self-efficacy.

Note. *N* = 289. LSE = linguistic self-efficacy; SRE = self-regulatory efficacy; DSE = delivery self-efficacy; PSE = performance self-efficacy. All item parameter estimates and latent variables correlations were significant (*p* < .001).

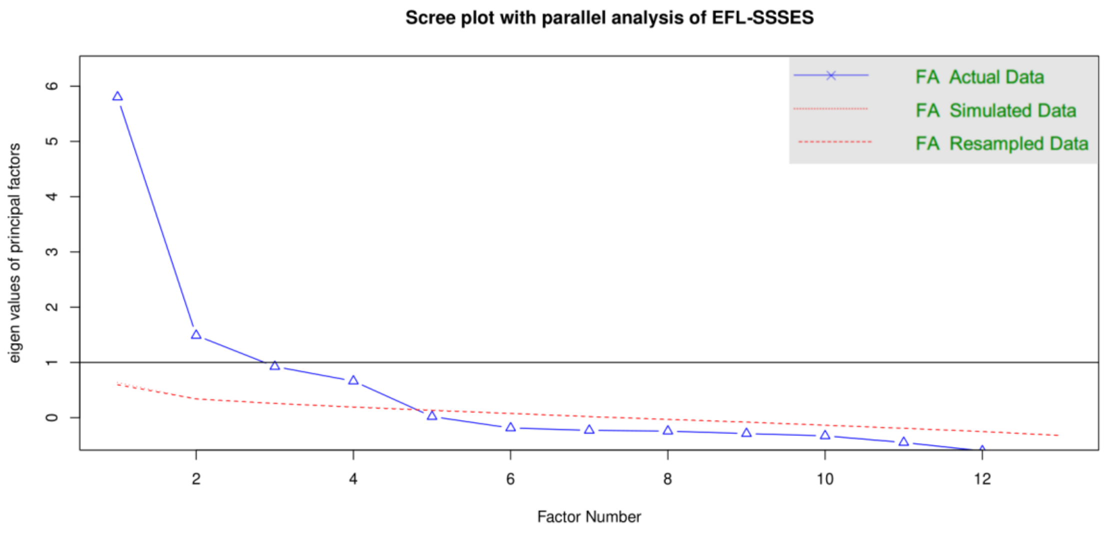

Fig 3. The scree plot with parallel analysis of EFL-SSSES.

Note. The point where the simulated eigenvalue (the red lines) meets the corresponding, real eigenvalue (the blue line) suggests that the number of factors.

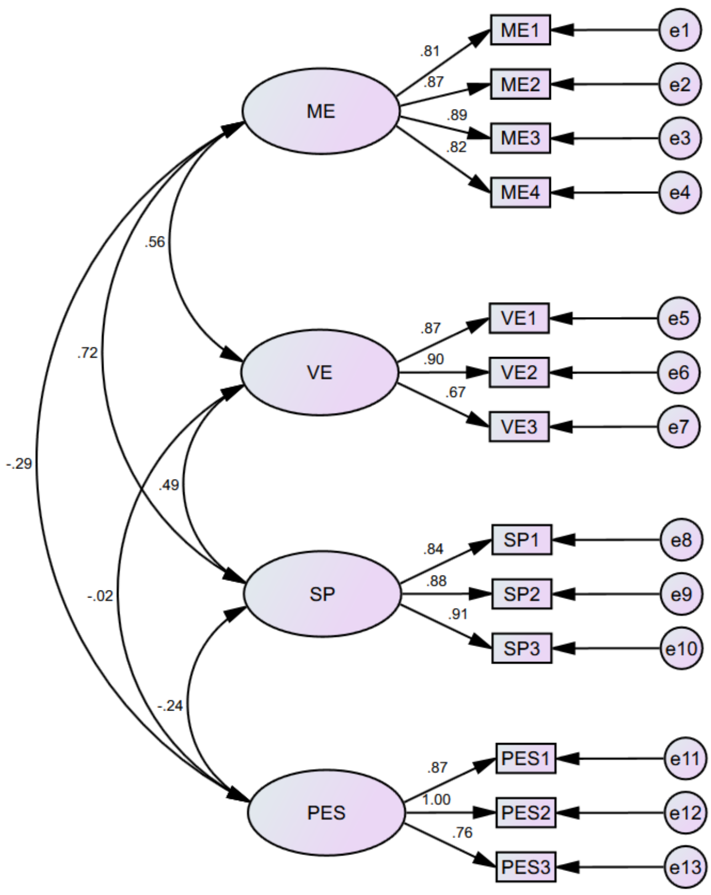

Fig 4. Four-factor correlated model of sources of EFL speaking self-efficacy.

Note. $N = 295$. ME = mastery experience; VE = vicarious experience; SP = social persuasion; PES = physiological and emotional states. All item parameter estimates and latent variables correlations were significant ($p < .001$).

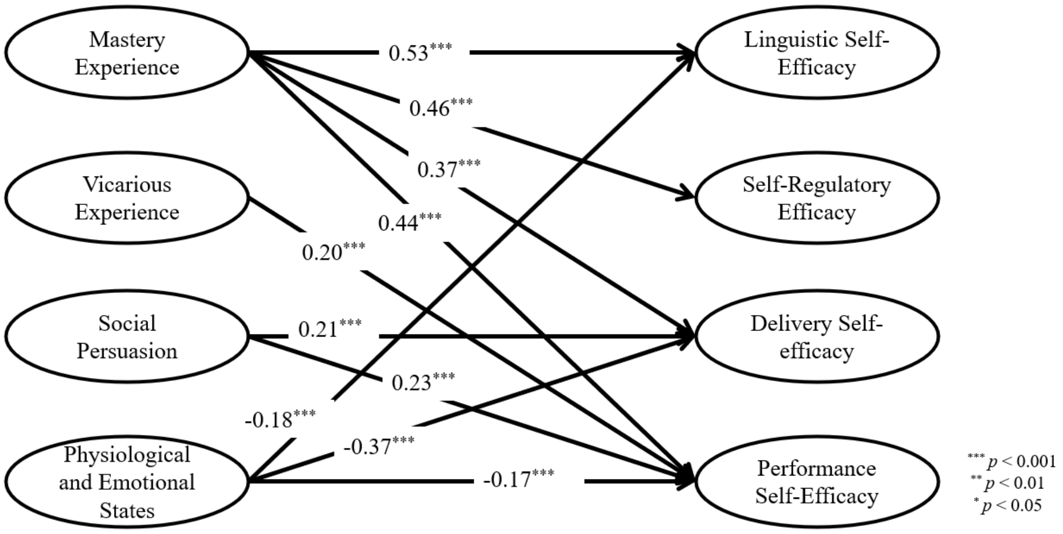

Fig 5. Path analysis and path coefficients of EFL speaking self-efficacy and its sources.

Comments

We recognize that you obtained ethical approval from your local ethics committee after you began the study: your ethics approval document is dated 08.12.2022, while participants were recruited beginning on 1st December 2022. For human subjects research, *PLOS ONE* requires that authors obtain ethics committee approval before beginning the study, and we do not accept retrospective ethical approval.

Jayati Bhattacharyya, PhD

Editorial Office

Response

Thank you for pointing out the typo in our manuscript. The actual data collection date falls after our ethics approval. That is, **11 December 2022**. We have corrected the typo in our revised submission. We fully understand the purpose of obtaining ethics approval which is to protect participants' rights, well-being, and privacy. That is why we sought ethics approval before initiating our research. We are sorry for the inconvenience caused during the process of our submission.

Authors
